# (Pro)Renin Receptor Decoy Peptide PRO20 Protects against Oxidative Renal Damage Induced by Advanced Oxidation Protein Products

**DOI:** 10.3390/molecules28073017

**Published:** 2023-03-28

**Authors:** Hui Fang, Teng Yang, Baolong Zhou, Xinxuan Li

**Affiliations:** School of Pharmacy, Weifang Medical University, Weifang 261053, China

**Keywords:** advanced oxidation protein products, (pro)renin receptor, PRO20, chronic kidney disease, renin-angiotensin system

## Abstract

Chronic kidney disease (CKD) is associated with advanced oxidation protein products (AOPPs). A recent study has shown that AOPP-induced renal tubular injury is mediated by the (pro)renin receptor (PRR). However, it is unclear whether the PRR decoy inhibitor PRO20 can protect against renal damage related to AOPPs in vivo. In this study, we examined the role of the PRR in rats with AOPP-induced renal oxidative damage. Male SD rats were subjected to unilateral nephrectomy, and after a four-day recuperation period, they were randomly divided into four groups (*n* = 6/group) for four weeks: control (CTR), unmodified rat serum albumin (RSA, 50 mg/kg/day via tail-vein injection), AOPPs-RSA (50 mg/kg/day via tail-vein injection), and AOPPs-RSA + PRO20 (50 mg/kg/day via tail-vein injection + 500 μg/kg/day via subcutaneous injection) groups. PRO20 was administered 3 days before AOPPs-RSA injection. Renal histopathology evaluation was performed by periodic acid–Schiff (PAS) staining, and biochemical parameters related to renal injury and oxidative stress biomarkers were evaluated. The expression of related indicators was quantified by RT-qPCR and immunoblotting analysis. In the results, rats in the AOPPs-RSA group exhibited higher levels of albuminuria, inflammatory cell infiltration, and tubular dilation, along with upregulation of oxidative stress, profibrotic and proinflammatory factors, and elevation of AOPP levels. Meanwhile, in the PRO20 group, these were significantly reduced. Moreover, the levels of almost all components of the renin-angiotensin system (RAS) and Nox4-dependent H_2_O_2_ production in urine and the kidneys were elevated by AOPPs-RSA, while they were suppressed by PRO20. Furthermore, AOPPs-RSA rats showed elevated kidney expression of the PRR and soluble PRR (sPRR) and increased renal excretion of sPRR. In summary, these findings suggest that PRR inhibition may serve as a protective mechanism against AOPP-induced nephropathy by inhibiting the intrarenal RAS and Nox4-derived H_2_O_2_ mechanisms.

## 1. Introduction

The incidence and prevalence of chronic kidney disease (CKD) are on the rise worldwide [1]. Advanced oxidation protein products (AOPPs) are key biomarkers associated with oxidative stress and inflammation, which can be initially detected at high levels in the plasma of CKD patients. AOPPs can be easily carried by albumin in vivo and are formed upon interactions between plasma proteins and chlorinated oxidants during oxidative stress [2]. AOPPs contribute to the development of diseases associated with oxidative stress such as CKD and diabetes [3]. A growing body of evidence from observations in cell cultures and animal studies have also demonstrated that AOPPs may contribute to the development and progression of CKD. For example, chronic exposure to AOPPs leads to the activation of the renin-angiotensin system (RAS) and the production of intracellular superoxide [4]. As proinflammatory mediators, intercellular adhesion molecules and vascular cell adhesion molecules are released when AOPPs stimulate endothelial cells in the kidney [5]. As a result of the long-term loading of AOPPs in streptozotocin-induced diabetic rats and unilateral nephrectomized rats, proinflammatory and profibrotic factors such as monocyte chemotactic protein-1 (MCP-1) and transforming growth factor-β1 (TGF-β1) are elevated [6]. Tumor necrosis factor-α (TNF-α), interleukin-1, and interleukin-6 are pro-inflammatory cytokines produced by monocytes following AOPPs [7]. Notably, AOPP-modified albumin had a 100-fold greater effect on ROS generation than unmodified albumin, resulting in more severe kidney damage [4]. There are various mechanisms through which AOPP-induced kidney damage may occur, including the induction of podocyte apoptosis, tubular RAS activation, endothelial cell oxidative stress, and inflammation [3]. However, it is still unclear whether the prorenin receptor (PRR, also known as ATP6AP2) is involved in the specific mechanism underlying the renal oxidative stress injury caused by AOPPs.

The PRR is a transmembrane receptor with a relative molecular mass of 39 kDa and belongs to the type I family of receptors. It specifically binds renin and prorenin, promoting angiotensin I generation as a component of the RAS, first cloned by Nguyen et al. [8]. Studies have shown that the PRR can regulate the RAS when it is induced by angiotensin II (Ang II) or DOCA-salt (deoxycorticosterone acetate) [9,10], Adriamycin, or albumin overload-induced nephropathy [11,12]. In fact, most of the studies have focused on demonstrating the PRR’s role in hypertension, cardiovascular and renal disease, and organ injury, as well as identifying the PRR as a therapeutic target to improve the RAS [13]. Reactive oxygen species (ROS) from NADPH oxidase 4 (Nox4) contribute to oxidative stress and fibrosis in the kidney [14,15]. Our recent study showed that soluble PRR (sPRR) mediates AOPP-induced renal epithelial cell injury via the augmented Nox4-derived hydrogen peroxide (H_2_O_2_) pathway [16]. As a consequence, the PRR might be able to regulate Nox4/H_2_O_2_ signaling during AOPP-induced nephropathy in rats.

PRR antagonist PRO20 binds to all PRR sites of prorenin, similar to HRP (L1PTDTASFGRILLKKMPSVR20), according to Li et al. [9]; a complete block of prorenin-induced phosphorylation of extracellular signal-regulated kinase 1/2 (ERK1/2) can be achieved by this compound, as well as an improvement in hypertension caused by DOCA salts [17]. In recent years, ample studies have validated the effectiveness of PRO20 in attenuating the hyperactivation of intrarenal RAS in hypertensive rats who are deficient in drinking water, high in potassium, high in fructose and salts, overloaded with albumin, and injected with Adriamycin, prorenin, and Ang II [9,18,19,20,21,22]. We have previously shown that the PRR controls Nox4-derived H_2_O_2_ synthesis in cultured human proximal tubular epithelial HK2 cells by regulating the intrarenal RAS, and these results implicate the PRR in regulating AOPP-induced oxidative stress and potential inflammation effects [16]. As of yet, it is unknown whether the PRR regulates in vivo. The correlation analyses were performed in order to investigate if PRR expression was associated with AOPP-induced rat kidney injury.

AOPPs causes renal injury in an underlying mechanism that has not yet been fully explored. Using a rat model of AOPP-induced nephropathy, we examined the potential pathogenic role of the PRR. The goal was to evaluate PRR antagonism with PRO20′s potential as a treatment for AOPP-induced renal oxidative stress injury and to investigate its mechanisms of action. It is important to note that PRO20 significantly minimalized AOPP-induced renal injury that was associated with suppressed intrarenal RAS activity. All these findings support PRR-dependent activation of the local RAS as a key driver of the disease process as well as PRO20 as a novel therapeutic option.

## 2. Results

### 2.1. PRO20 Improved Kidney Histological Damage Caused by AOPPs-rat Serum Albumin (RSA)

**Figure 1:**
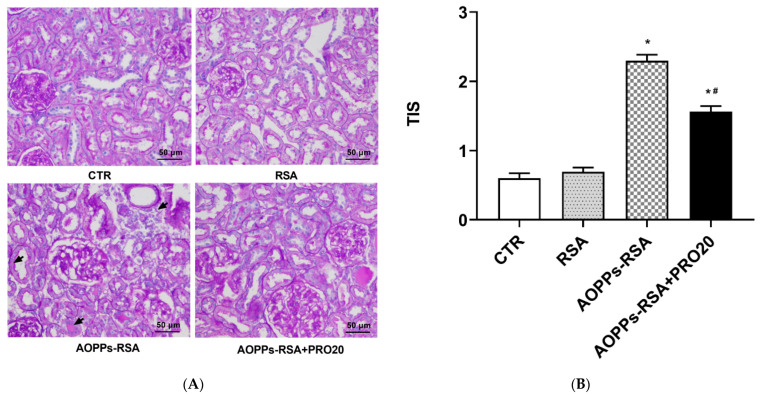
Histological evaluation of renal tissue and tubular injury. Notes: (**A**) Renal cortex sections were stained with periodic acid–Schiff (PAS) at the 4th week. (**B**) Quantification of tubulointerstitial injury scores (TIS) based on histology. At least 10 fields per rat were qualitatively analyzed (3 rats per group). The results were presented as means ± SEM. CTR, control; RSA, rat serum albumin; AOPPs, advanced oxidation protein products. * *p* < 0.05, compared to the CTR group; ^#^ *p* < 0.05, compared to the AOPPs-RSA group.

The kidneys in the AOPPs-RSA group showed features of tubular injury as indicated by the arrows, including tubular dilation, loss of the brush border, infiltration of inflammatory cells, and intratubular protein secretion (Figure 1A). In the AOPPs-RSA + PRO20 group, the histological damage caused by AOPPs-RSA decreased (Figure 1A), and the tubulointerstitial injury score (TIS) value was significantly lower than that in the AOPPs-RSA group (*p* < 0.05, Figure 1B).

**Figure 2:**
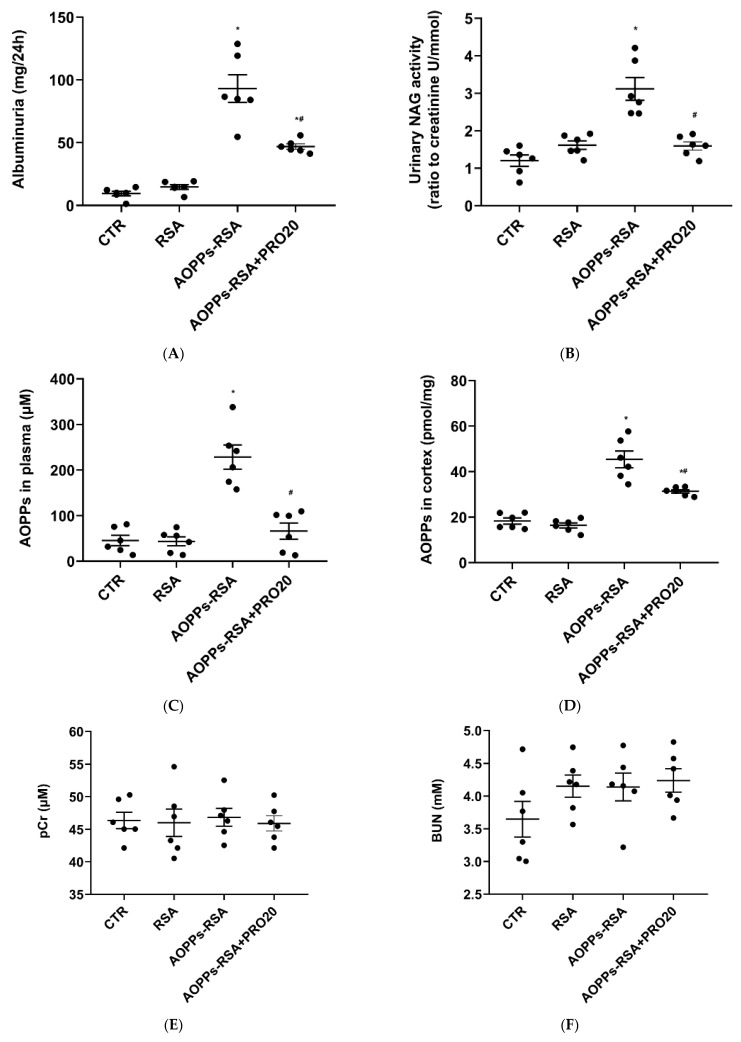
Effects of PRO20 on albuminuria (**A**), urinary NAG activity (**B**), AOPPs in plasma (**C**), AOPPs in renal cortex (**D**), pCr (**E**), and plasma BUN (**F**). Notes: NAG, N-acetyl-β-d-Glucosaminidase; pCr, plasma creatinine; BUN, blood urea nitrogen. The results are presented as means ± SEM. There were 6 rats per group. * *p* < 0.05, compared to the CTR group; ^#^ *p* < 0.05, compared to AOPPs-RSA group.

After four weeks of treatment, compared with the CTR group, the albuminuria and urinary N-acetyl-β-D-glucosaminidase (NAG) activity in the AOPPs-RSA group increased by ten-fold and three-fold, respectively (*p* < 0.05). However, compared with the AOPPs-RSA group, both of them were significantly reduced by 50% in the AOPPs-RSA + PRO20 group (*p* < 0.05). There was no significant difference in proteinuria and urinary NAG activity between the RSA and control groups (Figure 2A,B). Similarly, the AOPP levels in the plasma and renal cortex of the AOPPs-RSA group were significantly higher than those in the CTR group, but the AOPP levels in the AOPPs-RSA + PRO20 group were significantly lower than those of the AOPPs-RSA group by 60% and 30%, respectively (*p* < 0.05) (Figure 2C,D). Conversely, plasma creatinine (pCr) and blood urea nitrogen (BUN) remained relatively constant among the four groups (*p* > 0.05) (Figure 2E,F). As shown here, AOPPs-RSA-induced nephropathy triggers PRR-dependent mechanisms.

### 2.2. PRO20 Attenuated AOPPs-RSA-Induced Renal Inflammation and Fibrosis Markers

**Figure 3:**
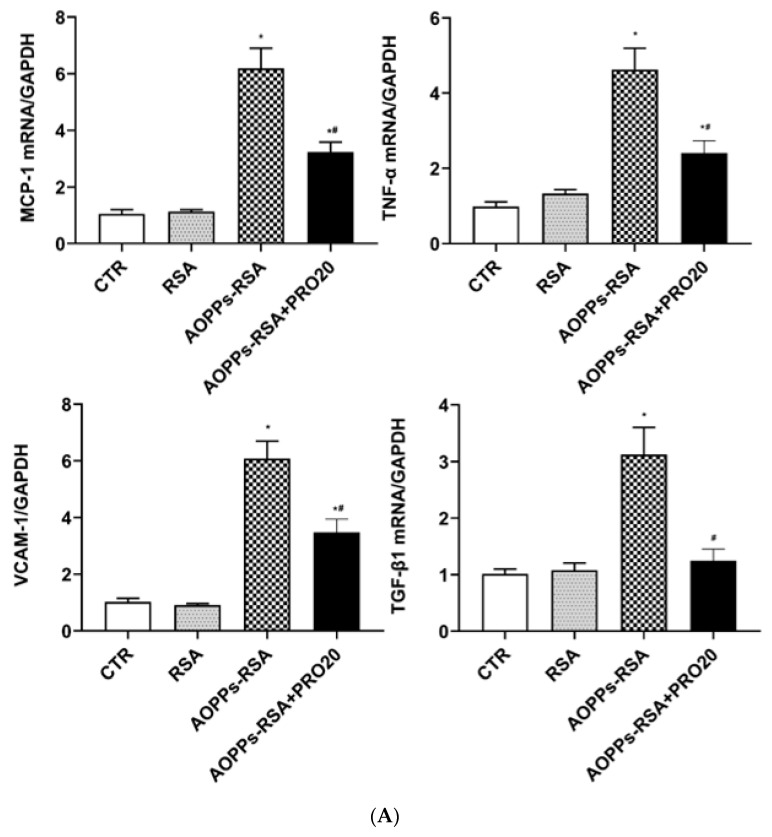

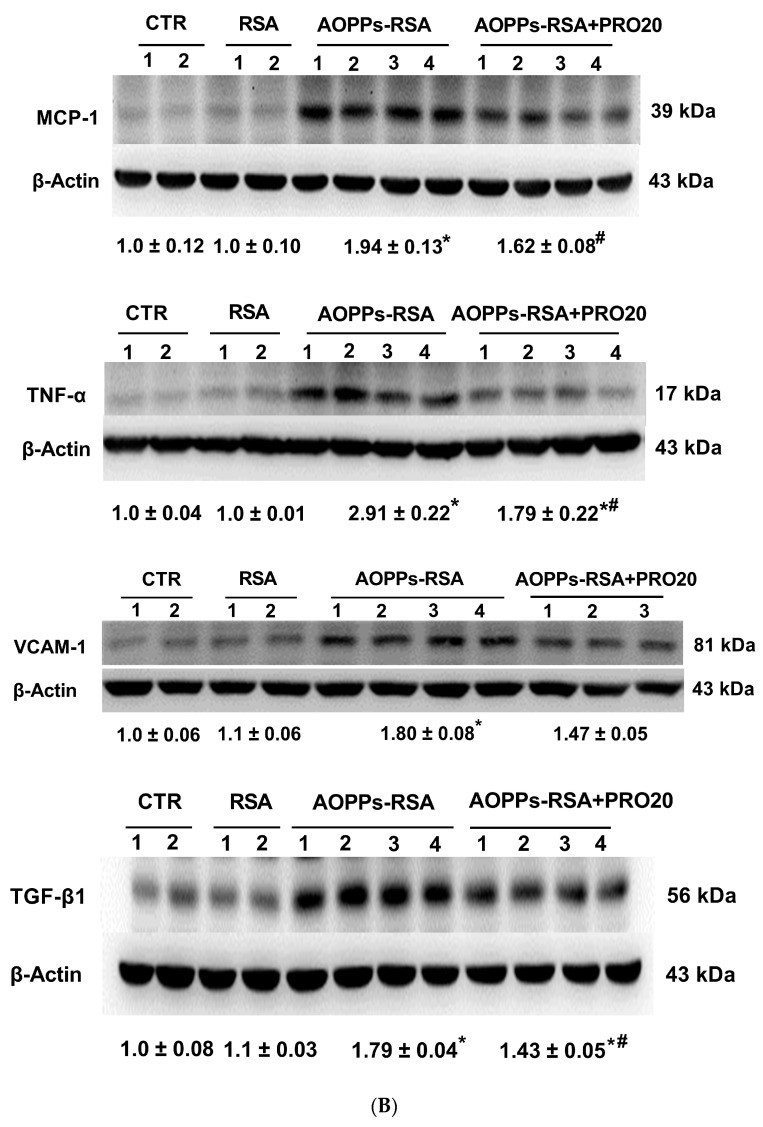
Administration of PRO20 reduced renal pro-inflammatory and fibrosis mediator secretion induced by AOPPs-RSA. Notes: (**A**) The mRNA expression levels of MCP-1, TNF-α, VCAM-1, and TGF-β1 in renal tissue were checked by RT-qPCR. All values were normalized against GAPDH expressions. (**B**) The protein levels of MCP-1, TNF-α, VCAM-1, and TGF-β1 were determined by immunoblotting analysis. The normalized quantitative data for β-actin are shown below each blot. All data are presented as mean ± SEM. There were 6 rats per group. * *p* < 0.05, compared to the CTR group; ^#^ *p* < 0.05, compared to AOPPs-RSA group. MCP-1, monocyte chemotactic protein-1; TNF-α, tumor necrosis factor-α; VCAM-1, vascular cell adhesion molecule-1; TGF-β1, transforming growth factor-β1.

The associations between inflammation and fibrosis and AOPPs-RSA-induced nephropathy are notable [23,24]. RT-qPCR and immunoblotting analysis were used to assess the mRNA and protein expression levels of pro-inflammatory factors (MCP-1, TNF-α, and VCAM-1) and pro-fibrotic factor (TGF-β1) in kidneys. As shown in Figure 3A, AOPPs-RSA significantly increased the mRNA levels of MCP-1 (~6.2-fold), TNF-α (~4.6-fold), VCAM-1 (~6.0-fold), and TGF-β1 (~3.1-fold); PRO20 treatment significantly decreased the mRNA levels of MCP-1 (~48%), TNF-α (~48%), VCAM-1 (~43%), and TGF-β1 (~60%) compared to the AOPPs-RSA group (*p* < 0.05). The protein levels of MCP-1, TNF-α, and TGF-β1 in the AOPPs-RSA rats were significantly higher than those in the CTR group (*p* < 0.05), but PRO20 treatment prevented these increases (*p* < 0.05, Figure 3B). In addition, we also found that the protein level of VCAM-1 was significantly higher in the AOPPs-RSA group than in the CTR group, and there was a slight reduction in VCAM-1 in the AOPPs-RSA + PRO20 group, but the differences were not statistically significant (*p* > 0.05, Figure 3B). Overall, these results strongly indicate that PRO20 offers renoprotection in AOPPs-RSA-induced renal injury.

### 2.3. PRO20 Attenuated AOPPs-RSA-Induced Renal Oxidative Stresses

**Figure 4:**
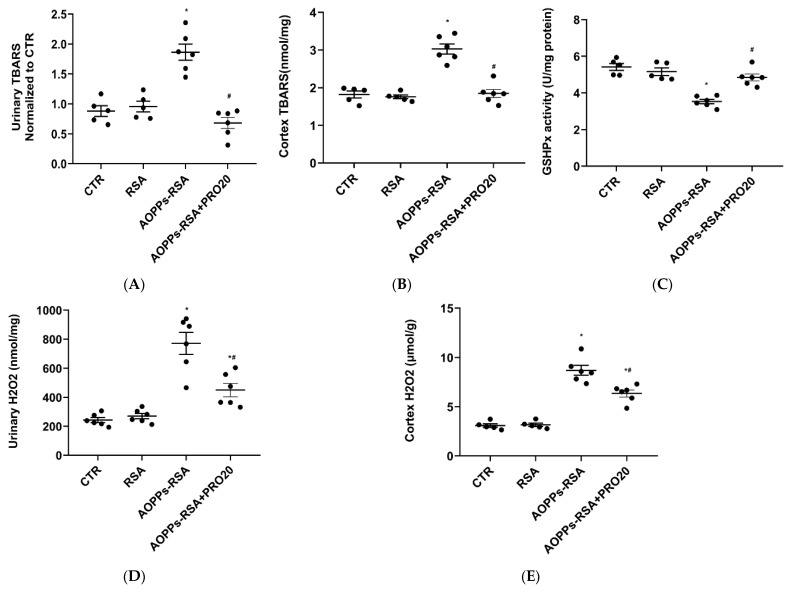

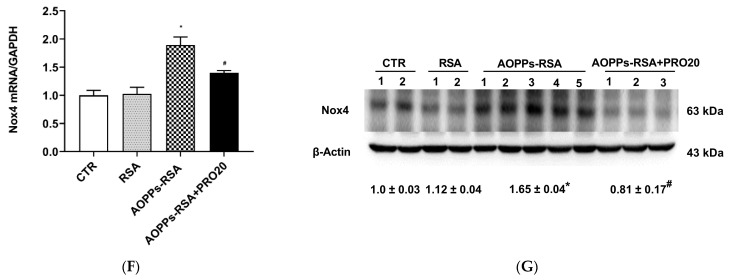
Administration of PRO20 reduced renal oxidative stresses induced by AOPP-RSA. Notes: (**A**) Urinary TBARS concentration; (**B**) renal cortex TBARS concentration; (**C**) renal cortex GSHPx activity; (**D**) urinary H_2_O_2_ content; (**E**) renal cortex H_2_O_2_ content; (**F**) mRNA levels of *Nox4* were determined by RT-qPCR. All values were normalized against GAPDH expressions; (**G**) protein expression of Nox4 was performed by immunoblotting. The normalized quantitative data for β-actin are shown below each blot. Results are presented as mean ± SEM. There were 6 rats per group. * *p* < 0.05, compared to the CTR group; ^#^ *p* < 0.05, compared to the AOPPs-RSA group.

In conjunction with inflammation, AOPPs-RSA also induced oxidative stress in CKD [3]. Therefore, we assessed the urinary thiobarbituric acid reactive substances (TBARS) level, which is an indicator of oxidative stress. There was a two-fold increase in urinary TBARS in the AOPPs-RSA group when compared to the CTR group, but this elevation diminished significantly in the AOPPs-RSA + PRO20 group, as well (*p* < 0.05, Figure 4A). As compared to the CTR group, the AOPPs-RSA group showed a 1.5-fold increase in renal TBARS levels. Figure 4B showed that PRO20 significantly inhibited this process. In comparison with the CTR group, GHx activity in the renal cortex was 30% lower in the AOPPs-RSA group, which was reversed by PRO20 treatment (Figure 4C). These results present evidence that PRR-dependent mechanisms contribute to oxidative stress in nephropathy induced by AOPPs-RSA.

Based on our previous study, AOPPs-RSA was shown to cause injury to kidney tubular epithelial cells by activating the PRR/Nox4/H_2_O_2_ pathway [16]. As expected, we found that AOPPs-RSA administration also significantly increased urinary and renal cortical H_2_O_2_ excretion, and PRO20 treatment antagonized these responses (*p* < 0.05) (Figure 4D,E). In addition, mRNA level of *Nox4* was significantly increased in the AOPPs-RSA group, whereas PRO20 treatment decreased this expression (*p* < 0.05) (Figure 4F). Similar results of Nox4 were also observed at the protein levels (*p* < 0.05) (Figure 4G). These indicated that Nox4 and H_2_O_2_ are triggered during nephropathy caused by AOPPs-RSA by PRR-dependent mechanisms.

### 2.4. PRO20 Inhibited AOPPs-RSA-Induced Activation of Renal Local RAS

**Figure 5:**
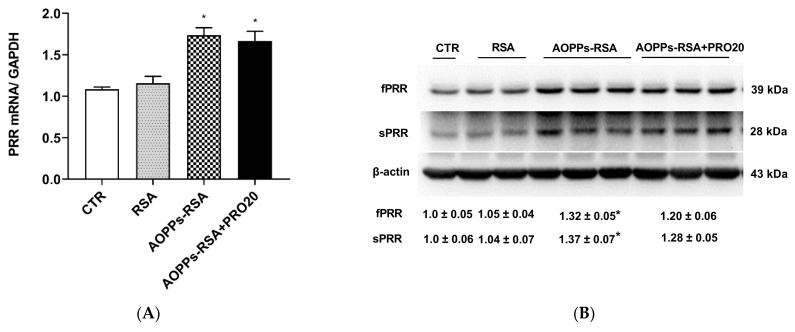

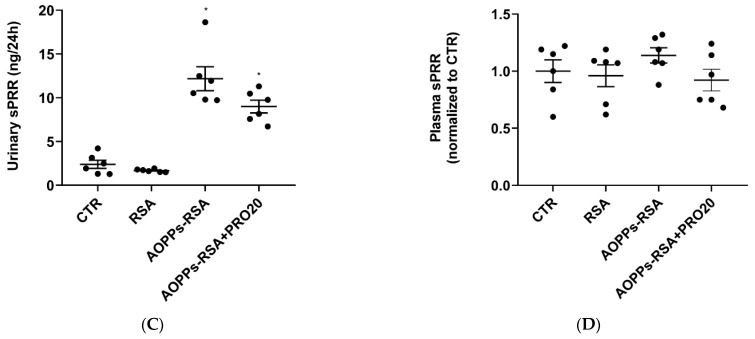
Expression analysis of the PRR/sPRR. Notes: (**A**) mRNA levels of PRR were measured by RT-qPCR. (**B**) Protein levels of fPRR and sPRR in renal cortex was assessed by immunoblotting. The normalized quantitative data for β-actin are shown below each blot. (**C**) Urinary sPRR excretion was analyzed with ELISA. (**D**) Plasma sPRR was analyzed with ELISA. Results are presented as mean ± SEM. *n* = 6 per group. * *p* < 0.05, compared to the CTR group. PRR: (Pro)renin receptor; fPRR: full-length PRR; sPRR: soluble PRR.

In rats subjected to unilateral nephrectomy, chronic AOPPs-RSA loading caused AOPPs-RSA deposition in proximal tubule cells and led to the activation of the RAS [3]. A recent study also showed that the PRR is a critical factor in regulating intrarenal RAS activation during albumin overload-induced nephropathy [11]. This study aims to determine whether the PRR can inhibit intrarenal RAS activation during AOPPs-RSA-induced nephropathy as a possible stimulus. As shown in Figure 5A–C, the renal PRR transcripts, full-length PRR (fPRR) and sPRR protein expressions, and urinary sPRR excretion were all elevated in the AOPPs-RSA group (1.7 times, 1.3 times, 1.4 times, and 5.1 times, respectively, *p* < 0.05) compared to the CTR group. However, PRO20 treatment did not significantly affect the expression of these proteins compared with the CTR group or the AOPPs-RSA group (*p* > 0.05). Conversely, plasma sPRR concentrations had no significant changes in these groups (Figure 5D). It is clear that intrarenal PRR/sPRR was activated by AOPPs-RSA.

**Figure 6:**
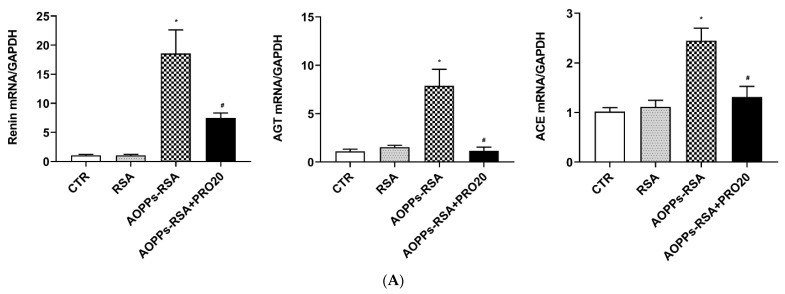

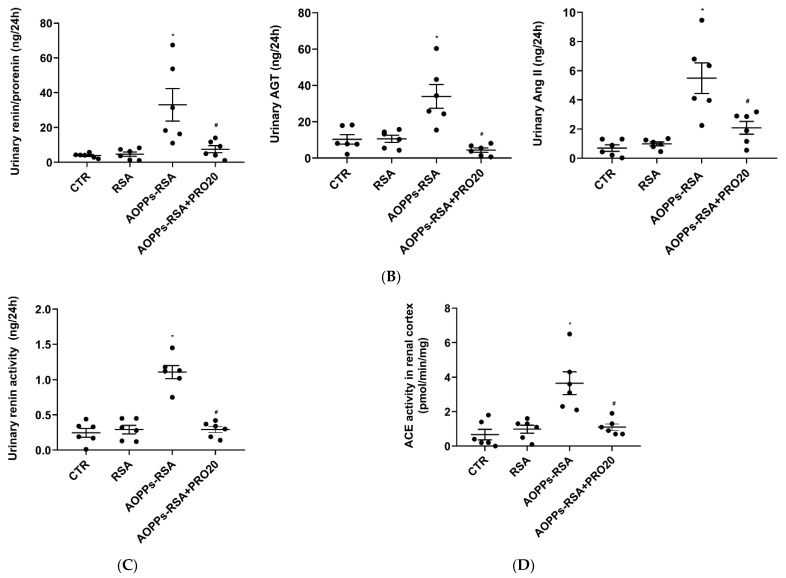
Analysis of the other intrarenal components of the RAS. Notes: (**A**) mRNA levels of *REN*, *AGT*, and *ACE* were determined by using RT-qPCR. All values were normalized against GAPDH expressions. (**B**) ELISA test was used to quantify urinary renin/prorenin, AGT, and Ang II levels. (**C**) Urinary renin activity. (**D**) ACE activity in renal cortex. Results are presented as mean ± SEM. There were 6 rats per group. * *p* < 0.05, compared to the CTR group; ^#^ *p* < 0.05, compared to the AOPPs-RSA group. RAS: renin-angiotensin system; REN: renin; AGT: angiotensinogen; ACE: angiotensin-converting enzyme; Ang II: angiotensin II.

In the AOPPs-RSA group, the mRNA expression levels of renal *REN* (renin), *angiotensinogen (AGT)*, and *angiotensin-converting enzyme (ACE)* were significantly increased, (*p* < 0.05), but decreased significantly after PRO20 treatment (Figure 6A). Similarly, the levels of urinary renin/prorenin, AGT, and Ang II in the AOPPs-RSA group were significantly higher than those in the CTR group (*p* < 0.05), but PRO20 treatment inhibited these levels (*p* < 0.05, Figure 6B). Furthermore, AOPPs-RSA significantly elevated urinary renin activity and renal cortex ACE activity, which were all attenuated by PRO20 treatment (*p* < 0.05, Figure 6C,D). According to the data presented here, the RAS is triggered by PRR-dependent mechanisms during nephropathy induced by AOPPs-RSA.

## 3. Discussion

CKD progression is mainly due to the abnormal activation of the intrarenal RAS. RAS inhibitor treatment has become the current standard of care for the preservation of CKD [25]. In this study, chronic perfusion of AOPPs-RSA in rats resulted in hyperactivity of RAS components in the kidney, including Nox4, MCP-1, TNF-α, VCAM-1, and TGF-β, which are associated with oxidative stress, pro-inflammatory and pro-fibrotic responses, and renal fPRR and sPRR expression, as well as urinary sPRR excretion. Notably, in rats treated with PRO20, AOPPs-RSA-induced renal pathology and albuminuria were prevented, as well as intrarenal RAS inhibition. The PRR was investigated in a rat model of AOPPs-induced nephropathy to identify the possible pathogenic mechanisms. Albuminuria, tubular dilation, brush-border loss, inflammatory cell infiltration, and interstitial fibrosis were some of the manifestations of renal injury from chronic AOPPs-RSA administration for 4 weeks. With PRO20, all the above symptoms and the renal function were significantly improved. A 4-week chronic AOPPs-RSA infusion also induced the expression of the PRR/sPRR in the kidneys and urinary sPRR excretion, while increasing renal ACE activity, renin activity, and urinary AGT and Ang II excretion. PRO20 as a PRR competitive peptide inhibitor inhibited RAS upregulation and improved urinary albumin and H_2_O_2_ production. Moreover, it also improved the protein expression of MCP-1, TNF-α, VCAM-1, TGF-β1, and Nox4 in renal tissue. In summary, the PRR was identified as the pathogenic component of AOPPs-RSA-induced renal oxidative stress injury in the animal experiment of our study, and PRO20 was shown to have potential therapeutic effects.

As evidenced by a growing body of research, the intrarenal RAS plays a role in the pathogenesis of hypertension and kidney failure [26]. The PRR was initially discovered on the basis of its ability to specifically bind to (pro)renin to increase enzyme activity by fivefold over non-receptor-bound renin. The PRR promotes the conversion of angiotensinogen to angiotensin I and is therefore involved in localizing RAS activation at the site where it is expressed [27,28]. HRP contains the sequence of an anchor site in (pro)renin required for PRR binding [17,29], which blocks the ability of the PRR to bind (pro)renin [30]. Although initial studies showed its benefits to diabetes and hypertension-induced nephropathy and retinopathy, later studies have come up with negative results [31,32,33]. A study of PRR inhibition by HRP in cultured vascular smooth muscle cells revealed that HRP failed to inhibit the activation of phosphorylated ERK1/2 by the PRR, which raised further concerns [33]. Unlike HRP, PRO20 contains all PRR binding sites on prorenin, completely inhibiting ERK1/2 phosphorylation after prorenin stimulation, and the NH2 terminus of this peptide is located close to the PRR binding domain (S149QGVLKEDVF158) on prorenin, preventing ERK1/2 phosphorylation after prorenin stimulation [9]. Previous studies [11,12] and the present study suggest that PRO20 interferes with the binding between the prosegment of prorenin and the PRR but has no effect on the expression of the PRR. Several rodent models with hypertension, fluid, and electrolyte imbalances, as well as dehydration have been validated by PRO20 [9,10,34,35,36,37]. Beyond this, we recognize that there are limitations to our experimental approach. Molecular docking remains a highly effective tool for analyzing molecular interactions. In future studies, we plan to incorporate molecular docking analysis to provide more detailed molecular insights into the interaction between prorenin and PRO20 in order to further understand their mutualistic relationship. Moreover, these studies suggested that the PRR may also be involved in regulating the intrarenal RAS. There is evidence that the PRR regulates the intrarenal RAS, but conflicting findings about their relationship have been reported. Saigo et al. demonstrated in their study that the overexpression of the tubular epithelial Atp6ap2 gene in mice led to hypertension independent of the RAS [18]. Moreover, overexpression of human PRRs did not affect the concentration of Ang II in tissues [38]. These observations raise questions about the role of the PRR as an important regulator of the local RAS. It is unclear if mice with tubular epithelial PRR overexpression display increased intrarenal or urine renin activity. Saigo et al. proposed that alternative intracellular renin, rather than conventional renin/prorenin, may participate in PRR signaling due to the physiological relationship between the PRR and renin/prorenin [18]. However, these are still not enough to believe that the PRR controls a variety of intracellular signal pathways and activates mitogen-activated protein kinase signals, the Wnt/ β- Catenin signal, and the cyclooxygenase-2/prostaglandin E2 signal [26]. In this study, we examined various intrarenal RAS parameters in rats treated with AOPPs-RSA or/and PRO20. Administration of AOPPs-RSA increased urinary AGT, sPRR, total renin levels, and renal and renin activity. Concomitantly, renal ACE activity and urinary Ang II levels also increased. PRO20 attenuated a number of RAS components within the renal cortex during AOPPs-RSA nephropathy, thereby providing further evidence that the PRR and intrarenal RAS are associated. These findings revealed a novel mechanism underlying the pathogenesis of AOPPs-RSA-mediated kidney injury.

AOPPs-RSA is regarded as an important marker of oxidative stress in tissues. The inhibition of oxidative stress is a promising pharmacological approach to prevent the effects of AOPPs-RSA on renal injury [39]. The presence of NOX is one of the significant causes of oxidative stress in diabetic nephropathy and CKD [40,41]. Reactive oxygen species (ROS) generated by Nox are involved in the pathogenesis of a variety of kidney diseases [42]. Nox4 is the major NADPH isoform in the kidney that controls physiological functions [43]. Nox4 is highly expressed in proximal tubules but relatively low in glomeruli [44]; its primary function is to produce H_2_O_2_, an antioxidant required for certain kidney functions [45]. For instance, Nox4 is involved in the PRR-stimulated expression of Na+ channels in epithelial duct cells (via H_2_O_2_ generation) [46]. In cultured renal proximal tubular epithelial cells, AOPPs-RSA activates Nox4 and produces ROS [3]. The Nox4 gene responds to various stresses such as ischemia, hypoxia, cytokines, and mechanical stress [47]. The present study aimed to investigate the potential of Nox4 as a therapeutic target for AOPPs-RSA-induced kidney disease by examining its role in the pathogenesis of AOPPs-RSA. In vitro, we observed that AOPPs-HSA upregulated Nox4 expression in human HK-2 cells [16]. Furthermore, the present study demonstrated that AOPPs-RSA can upregulate NOX4 expression and generate H_2_O_2_ in AOPPs-RSA rats, suggesting that AOPPs-RSA/NOX4 are capable of forming a positive feedback loop via H_2_O_2_. Nox4 and H_2_O_2_ mRNA and protein levels in renal tissues and urine were examined to determine whether PRO20 protects against AOPPs-RSA. As a result of PRO20 treatment, we observed a decrease in Nox4 expression in renal tissue and a decrease in H_2_O_2_ generation in renal tissue and urine in AOPPs-RSA-treated rats. These studies found that PRO20 reduced oxidative stress in kidney cells when exposed to AOPPs-RSA.

The Ang II complex contributes to the pathogenesis of CKD through a complex mechanism involving oxidative stress [48,49] and upregulates Nox subunits in kidney injuries [50]. After PRO20 was administered, Ang II levels in urine and ACE transcript levels in kidneys were decreased, demonstrating the inhibition of intrarenal RAS activation. In AOPPs-RSA nephropathy, we hypothesized that PRO20 inhibits oxidative stress by inhibiting intrarenal RAS activation. Similar to that, our previous findings demonstrated that AOPPs-RSA enhances oxidative stress by generating Nox4-derived H_2_O_2_ and RAS by PRR-dependent mechanisms in vitro [16]. Moreover, oxidative stress was reported to be mediated by the PRR in neuronal cells both in Ang II-dependent and -independent conditions [51]. A PRR-dependent H_2_O_2_ derived from Nox4 was required for prorenin to significantly stimulate ENaC activity, although not the RAS [46]. The above indicated that PRO20 was found to inhibit both Nox4-derived H_2_O_2_ and RAS activity in AOPPs-RSA nephropathy by inhibition of the PRR-dependent process. However, further studies on AOPPs-RSA-induced kidney injury are required to determine whether PRR and Nox4 have a direct interaction.

The fPRR is consumed by proteases to produce sPRR, which contains the N-terminal extracellular domains of the PRR [22,52,53,54], a C-terminal intracellular domain of 8.9 kDa, and an ATPase-associated truncated protein [55]. Like fPRR, sPRR has a crucial role in various physiological and pathological processes [56], such as the activation of tissue RAS [57], regulation of water balance [58], hypertension [59], and tubular injury [60]. It has been demonstrated that excess albumin can activate the expression of sPRR in HK-2 renal proximal tubular epithelial cells [16]. Moreover, AOPP-modified albumin can more efficiently generate sPRR by cleaving the PRR from its full-length form in vitro and in vivo than unmodified albumin [16,52]. In cultured HK-2 cells, AOPPs-HSA significantly increased the expression of sPRR at 0.5% of the native RSA concentration [16]. Additionally, loading AOPPs-RSA with 50 mg/kg/day for 4 weeks induced the same levels of urinary sPRR secretion as loading with 5 g/kg/day of albumin for 7 weeks [11]. Our study also found that inhibiting the production of sPRR derived from Site-1 protease (S1P) significantly inhibited activation of the local RAS, inflammatory responses, and cellular damage induced by AOPPs-HSA or albumin overload, whereas sPRR-His reversed the effects of S1P inhibition [16,52]. However, further investigations are necessary to explore the possibility of whether AOPPs can modulate the activity or expression of S1P and to elucidate its relationship to the pathogenesis of AOPP-induced nephropathy in vivo. The inhibition of sPRR generation may present a promising therapeutic strategy to treat AOPP-induced renal damage.

**Figure 7:**
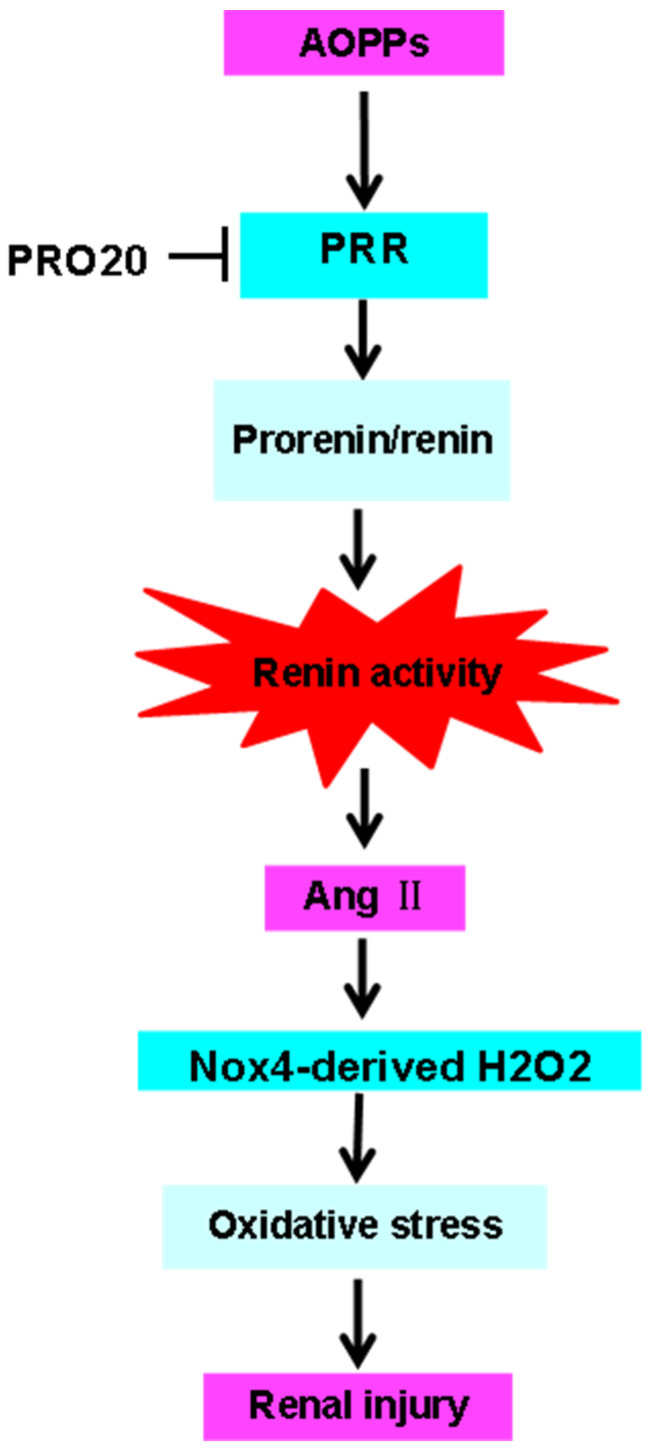
Diagram illustrating the action mechanism of AOPPs-RSA. AOPPs-RSA induces activation of the RAS, which leads to higher renin activity and Ang II release that activates Nox4-dependent H_2_O_2_ production and eventually results in renal injury. This process was abolished by PRO20. Abbreviations: AOPPs, advanced oxidative protein products; PRR, renin/prorenin receptor; Ang II, angiotensin II; Nox4, NADPH oxidase 4; H_2_O_2_, hydrogen peroxide.

Despite some findings, the present study has several limitations. One of which is that it is not clear whether the decoy inhibitor PRO20 binds to the extracellular domain of the PRR, such as the full-length or soluble form, which excludes the prosegment of prorenin from the interaction. Further experimentation is necessary to determine this. Although PRO20 is shown to be a highly effective and specific PRR inhibitor based on these results, additional studies are necessary to fully evaluate the peptide inhibitor more thoroughly. In addition, as a result of our study, the chronic systemic AOPPs-RSA activation of rat PRR in vivo has been well-established. As noted by the study, PRO20 has also been found to protect against AOPPs-RSA-induced nephropathy by inhibiting the RAS and oxidative stress-related protein Nox4 and H_2_O_2_ production (Figure 7). It will also be necessary to conduct subsequent studies, specifically those that utilize tissue-specific PRR knockout animal models, to fully address PRO20′s pharmacological actions.

## 4. Materials and Methods

### 4.1. Preparation and Measurement of AOPPs-RSA

The AOPPs-RSA was prepared as described previously [4,61]. The AOPPs in plasma were centrifuged for one hour at 4 °C at 10,000× *g* to eliminate the interference of lipid turbidity on light absorption. Quantification of AOPPs in homogenized kidney tissue was performed by using spectrophotometry as previously described [61]. The absorbance of the reaction mixture was immediately measured at 340 nm on a microplate reader (BioRad, Tokyo, Japan). AOPP content in AOPPs-RSA was 4.93 ± 0.60 nmol/mg, while that in unmodified RSA was 0.20 ± 0.02 nmol/mg.

### 4.2. Animals

Adult male Sprague–Dawley (SD) rats aged 7 to 8 weeks with 160 to 180 g body weight were purchased from Pengyue Laboratory Animal Breeding Co., Ltd. (Jinan, China). Rats were housed 3–4 per cage under environmentally controlled conditions (22 °C and 12/12 h light/dark) with ad libitum access to food and drinking water. All animal experiments were approved by the Animal Care and Use Committee of Weifang Medical University (license number: 2022SDL227).

### 4.3. Experimental Design and Treatment Groups

**Figure 8:**
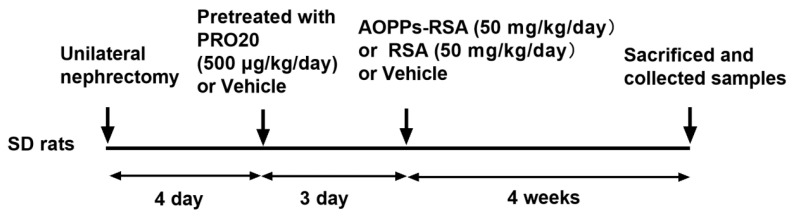
Graphical workflow of the experimental design. Notes: SD rats were administered CTR, RSA, AOPPs-RSA, or AOPPs-RSA in combination with PRO20; PRO20 was subcutaneously administered three times (7:00, 15:00, 23:00, respectively) every day. AOPPs-RSA or RSA were administered by tail vein injection once daily for 4 weeks. Administration of PRO20 started 3 days prior to administration of AOPPs-RSA or RSA. AOPPs: advanced oxidation protein products; RSA: rat serum albumin.

After one week of adaptive feeding, there was a unilateral left nephrectomy performed on all 24 rats, followed by a 4-day recuperation period. Afterward, rats were randomly divided into four groups (*n* = 6/group) for 4 weeks, including the control (CTR) group (tail vein injection of phosphate-buffered saline, PBS + subcutaneous injection of normal saline, NS); RSA group (tail vein injection of RSA 50 mg/kg + subcutaneous injection of NS); AOPPs-RSA group (tail vein injection of AOPPs-RSA 50 mg/kg + subcutaneous injection of NS); and AOPPs-RSA + PRO20 group (tail vein injection of AOPPs-RSA 50 mg/kg + subcutaneous injection of PRO20 dissolved in saline, 500 μg/kg) (Figure 8). The PRR decoy peptide PRO20 (LPTDTASFGRILLKKMPSVR; purity, 98%) was synthesized by Huada Gene Company (Beijing, China). PRO20 was delivered 3 days before AOPPs-RSA injection.

According to the above administration and dosage, tail vein injection was performed once a day, and subcutaneous injection was performed three times a day (7:00, 15:00, and 23:00, respectively) for all groups. At the 4th week, a 24-h urine sample was collected from each rat in a metabolic cage. After the 4-week experiments, rats were euthanized with 2–3% soflurane, and the kidneys were quickly removed and frozen in liquid nitrogen. Then, 3~5 mL blood samples were immediately obtained from the inferior vena cavas into EDTA anticoagulant tubes and centrifuged (1000 rpm for 10 min) to collect plasma.

### 4.4. Determination of Biochemical Parameters

Urine samples were centrifuged at 10,000 rpm for 5 min. The urinary albumin concentration was measured using the urinary albumin assay kit (Nanjing Jiancheng Bioengineering Institute, Nanjing, China). Urine NAG activity was quantified using a commercial NAG assay kit from Nanjing Jiancheng Bioengineering Institute. In accordance with the manufacturer’s instructions, pCr and BUN levels were measured using colorimetric detection kits (Nanjing Jiancheng Bioengineering Institute, Nanjing, China).

### 4.5. Histopathological Assessment

The Paraffin-embedded kidneys were fixed in 10% formalin for 24 h. Tissue sections that were 5 μm thick were used for periodic acid–Schiff (PAS) staining. Microphotographs were obtained using a Leica microscope (Leica microsystems Co., Wetzlar, Germany). Image analysis was performed with ImageJ software (version 146, National Institutes of Health, Bethesda, MD, USA). The severity of renal tubular damage was assessed according to the tubular damage score described previously [62]. TIS was indicated by tubular dilation, protein casts, inflammatory cell infiltration, and brush-border loss (the higher the score, the worse the injury). To semi-quantify the tubulointerstitial area, 10 areas of the renal cortex were randomly selected. The percentages of each area showing histological changes were estimated, including loss of brush border, tubular dilation, protein cast formation, and inflammatory cell infiltration. The score was assigned as follows: (0), normal; (1), involvement of <10% of the area; (2), involvement of 10 to 30% of the area; (3), involvement of 30 to 50% of the area; (4), involvement of >50% of the area. The TIS was calculated as: [(0 × n0) + (1 × n1) + (2 × n2) + (3 × n3) + (4 × n4)]/20.

### 4.6. Biochemical Analysis of AGT, ANG II, sPRR, ACE Activity, and Renin Activity

The total urinary renin/prorenin levels were measured by using a commercial ELISA kit (RPRENKT-TOT, Invitrogen, Carlsbad, CA, USA). The levels of urinary AGT and Ang II were measured by ELISA kit (SEA797Ra; CEA005Ra, Cloud-Clone Corp., Houston, TX, USA). Plasma and urine levels of sPRR were measured using the sPRR assay kit (Immuno-Biological Laboratories, Gunma, Japan). The ACE activity of renal cortex tissue was measured as previously described [23]. Briefly, an aliquot sample was incubated with hippuryl-histidyl-leucine (Sigma-Aldrich, St. Louis, MO, USA), a synthetic substrate specific to ACE. Incubation with o-phthaldialdehyde transformed liberated His-Leu into a fluorescent product. After the excitation and emission wavelengths of 364 and 486 nm, the amount of His-Leu was determined by fluorescence using Dynex technologies. The renin activity assay was performed as described previously, and renin activity was determined by the delta value of Ang I generation using an Ang I EIA kit (S-1188, Bachem, Bubendorf, Switzerland) [11].

### 4.7. Measurement of TBARS Concentration, H_2_O_2_ and GSHPx Activity in Urine and Renal Cortex

TBARS in urine and the renal cortex were measured using TBARS assay kit (10009055; Cayman Chemical, Michigan, MI, USA) for measuring malondialdehyde levels [11]. The enzyme activity of Glutathione peroxidase (GSHPx) in the renal cortex was assessed using a GSHPx assay kit (Nanjing Jiancheng Bio-Engineering Institute Co., Ltd., Nanjing, China) based on Maral et al. [63]. The H_2_O_2_ concentrations in urine and the renal cortex were measured by the ROS-Glo H_2_O_2_ assay kit (Promega, Madison, WI, USA).

### 4.8. Reverse Transcription Quantitative PCR (RT-qPCR)

Total RNA was extracted from the homogenized renal tissue of six individuals per group using TRIzol reagent (Cwbio, Beijing, China). Reverse transcription and total RNA isolation was carried out according to the previously described methodology [52]. DNase free RNAse treatment of the RNA samples was performed following reverse transcription by Ambion. Transforming RNA into cDNA was performed using a Transcriptor First Strand cDNA Synthesis Kit (Roche, Basel, Switzerland). The primers were designed using Primer3 software (http://flypush.imgen.bcm.tmc.edu/primer/primer3_www.cgi accessed on: 20 February 2023) (Table 1). Light Cycler 480 Real-Time PCR System was used to perform qPCR using Roche’s SYBR Green Master Mix (Roche). qPCR reaction systems and conditions are shown in Table 2. The relative mRNA expression levels were normalized with respect to the expression levels of GAPDH by the 2^−ΔΔCt^ method.

### 4.9. Immunoblotting Analysis

Immunoblotting analysis was performed as described by Wang et al. [64]. Kidney samples of 50–200 mg from each group were ground with grinding rods and lysed with RIPA buffer (Beyotime, China) containing PMSF, and the mixture was centrifuged at 12,000 g for 10 min at 4 °C. All samples were adjusted with RIPA buffer to achieve equal concentrations of protein in the supernatant using an Enhanced BCA Protein Assay Kit (Beyotime, Beijing, China). Then, 30 g of protein per sample was separated by 10% SDS-PAGE and transferred to nitrocellulose membranes (PVDF). The membrane was blocked with 5% skim milk for 1 h and then sequentially incubated overnight at 4 °C with the primary antibody. The following antibodies were used as primary antibodies: anti-PRR (HPA003156, Sigma-Aldrich, St. Louis, MO, USA), anti-TGF-β1 (SAB1305447, Abcam, Cambridge, UK), anti-TNF-α (sc-52746, Santa Cruz Biotechnology Inc., Santa Cruz, CA, USA), anti-MCP-1 (A00056-4, Boster Biological Technology, Pleasanton, CA, USA), and anti-VCAM-1 (ab134047, Abcam). Then, secondary antibodies were added after washing the membranes with TBST. The plots were visualized using enhanced chemiluminescence (Thermo Fisher Scientific, Waltham, MA, USA) and analyzed by the ImageJ software [9].

### 4.10. Statistical Analysis

All data are expressed as mean ± standard error of mean (SEM). One-way analysis of variance (ANOVA) with Bonferroni correction was used to determine differences between groups. Statistical analysis was performed using GraphPad Prism version 6.0 (GraphPad Software, San Diego, CA, USA) with a significance level set at *p* < 0.05.

## 5. Conclusions

The present study investigated the potential pathogenic role of the PRR, a novel member of the RAS, in an AOPPs-RSA-induced nephropathy rat model. The findings show evidence that the PRR is activated by AOPPs-RSA in parallel with enhancement of the intrarenal RAS. Crucially, PRR antagonism with PRO20 significantly attenuated AOPPs-RSA-induced renal injury associated with the suppressed intrarenal RAS. Overall, these findings suggest that PRO20 can potentially treat CKD. On the basis of the results of our study, future clinical trials can be designed rationally.

## Figures and Tables

**Table 1:** Primers used for RT-qPCR analysis.

Target Gene	Forward Primer Sequence (5′–3′)	Reverse Primer Sequence (5′–3′)
*MCP-1*	TAGCATCCACGTGCTGTCTC	CAGCCGACTCATTGGGATCA
*TNF-α*	CGTCAGCCGATTTGCCATTT	TCCCTCAGGGGTGTCCTTAG
*VCAM-1*	GGCTCGTACACCATCCGC	CGGTTTTCGATTCACACTCGT
*TGF-β1*	CTCAACACCTGCACAGCTCC	AGTTGGCATGGTAGCCCTTG
*PRR*	ATCCTTGAGACGAAACAAGA	AGCCAGTCATAATCCACAGT
*REN*	GATCACCATGAAGGGGGTCTCTGT	GTTCCTGAAGGGATTCTTTTGCAC
*AGT*	AGCATCCTCCTTGAACTCCA	TGATTTTTGCCCAGGATAGC
*ACE*	GAGCCATCCTTCCCTTTTTC	CCACATGTTCCCTAGCAGGT
*Nox4*	TGTGCCGAACACTCTTGGC	ATATGCACGCCTGAGAAAATA
*GAPDH*	AGACAGCCGCATCTTCTTGT	TTCCCATTCTCAGCCTTGAC

**Table 2:** RT-qPCR reaction system and conditions.

Items	Details
Reaction system	FastStart Universal SYBR Green Master (ROX): 5.0 μL; forward and reverse primer (1.5 µM): 3.0 μL; cDNA: 2.0 μL.
Reaction conditions	95 °C for 10 min; 40 cycles of 95 °C for 10 s and 60 °C for 30 s; followed by a melting curve.

## Data Availability

Not applicable.

## Sample Availability

Samples of the compounds PRO20 are not available from the authors.
